# Use of focused intensive care echo in the diagnosis of primary cardiac angiosarcoma

**DOI:** 10.1002/ccr3.3332

**Published:** 2020-09-17

**Authors:** Jasmine Medhora, Graham Wilson, Andrew Ronald, Lee Allen

**Affiliations:** ^1^ Intensive Care Department Aberdeen Royal Infirmary Aberdeen UK; ^2^ Anaesthetics Department Aberdeen Royal Infirmary Aberdeen UK

## Abstract

Focused Intensive Care Echo can aid diagnosis in complex patients where initial investigations are non‐diagnostic. FICE is an essential skill for critical care physicians, and bedside echocardiography should be a standard investigation for all critical care patients.

## Graphical abstract

FICE can aid diagnosis in complex patients where initial investigations are non‐diagnostic. FICE is an essential skill for critical care physicians, and bedside echocardiography should be a standard investigation for all critical care patients.

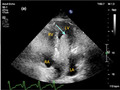

## 1 INTRODUCTION

Focused Intensive Care Echo (FICE) is recommended in critically unwell patients to assess hemodynamic status. In certain circumstances, FICE can also be used as a diagnostic tool. We present a case where FICE identified a large left atrial mass in an intensive care patient with acute hypoxia and thromboembolic sequelae.

The incidence of primary cardiac tumors is less than 1%,^1^ and angiosarcoma is the most common malignant primary cardiac tumor in adults.^1^ Cardiac Angiosarcoma can present with signs of heart failure, constitutional symptoms such as weight loss and systemic embolic events.^2^ Echocardiography is crucial for diagnosis of these tumors.^2^, ^3^

Traditionally, it has always been considered that transthoracic echocardiography (TTE) by a fully accredited practitioner is required to pick up rare cardiac tumors. While it is true that a full TTE allows detailed characterization of these tumors, the initial identification of an abnormal cardiac mass can be achieved with a basic focused echocardiography tool such as Focused Intensive Care Echo (FICE). The FICE training program was designed by the Intensive Care Society in collaboration with the British Society of Echocardiography (BSE).^4^ FICE can be performed by any clinician in critical care who has the accreditation. Using the FICE protocol, practitioners specifically look for left and right ventricular pathology, pericardial fluid, and pleural fluid to give real‐time assessment of hemodynamics, cardiac function, and filling. However, if FICE practitioners develop a high level of scanning ability, other pathology can also be identified. Ultimately, this means that FICE should be regarded as a non‐invasive diagnostic tool which should be available to all critical care clinicians as it can influence patient management significantly.

## 2 CASE PRESENTATION

A 55‐year‐old woman presented to the emergency department having woken up acutely short of breath. Prior to admission, she had been under investigation for chronic diarrhea, weight loss, and lethargy. She had no prior cardiac history. Plain chest radiograph demonstrated bilateral pulmonary infiltrates, and arterial blood gas analysis showed type 1 respiratory failure. The initial working diagnosis was of atypical pneumonia, and endo‐tracheal intubation was performed due to worsening hypoxemia. During transfer to critical care, the patient was noted to have atrial ectopic beats on electrocardiograph (ECG) monitoring.

Over the next 24 hours, the patient's respiratory function improved; however, she was found to have new right hemiparesis and a computed tomography (CT) brain scan diagnosed an acute ischemic infarction in the left parietal lobe. Aspirin was started, and the patient was extubated. Following extubation, her respiratory function began to deteriorate again, and the atypical pneumonia screen was negative. Pulmonary edema was therefore considered as a possible diagnosis, and so, a FICE scan was performed at the bedside. This revealed a large mass in the left atrium which prompted a CT chest, abdomen and pelvis scan and transesophageal echocardiography (TOE) as part of the preoperative patient work‐up for cardiothoracic surgery.

**FIGURE 1:**
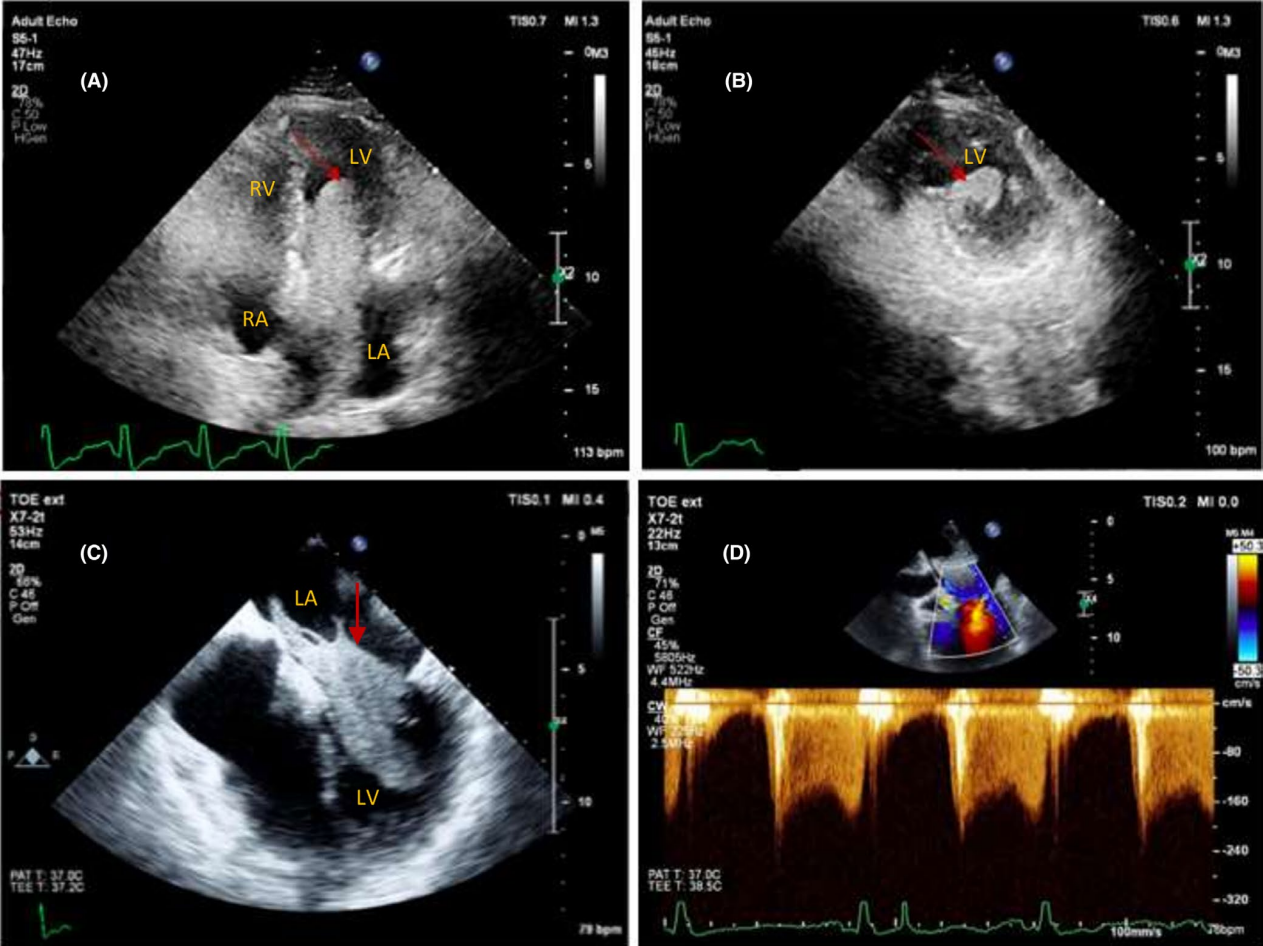
A, Focused Intensive Care Echo: apical four‐chamber view. Arrow shows left atrial mass prolapsing through mitral valve. B, Focused Intensive Care Echo: parasternal short‐axis view. Arrow indicates left atrial mass at level of papillary muscles. C, Transesophageal Echo: mid‐esophageal four‐chamber view. Arrow shows left atrial mass. D, Transesophageal echo with Doppler illustrating functional mitral stenosis due to the left atrial mass. The mitral valve area measures approx. 1.6 cm^2^, and the mean gradient was >5 mm Hg

The FICE scan demonstrated a large left atrial mass extending into the left ventricle through the mitral valve. This was best appreciated on the apical four‐chamber view as demonstrated in Figure 1A. Further imaging performed by a specialist transesophageal echocardiographer confirmed a 10 cm × 3 cm cystic mass (Figure 1C) resulting in functional mitral stenosis (Figure 1D) and acute pulmonary edema. Coronary angiography showed no atherosclerotic disease, and the patient was immediately taken to the operating room for excision of the mass. The postoperative recovery period was uncomplicated, and the patient was extubated and went on to rehabilitate. Histological analysis showed a high‐grade, sarcomatoid malignancy comprising sheets of epithelioid cells with large, markedly pleomorphic, and irregular nuclei with coarse chromatin and prominent nucleoli. Sections of cardiac muscle were seen in some of the fragments but did not appear directly infiltrated. Findings were consistent with angiosarcoma.

**FIGURE 2:**
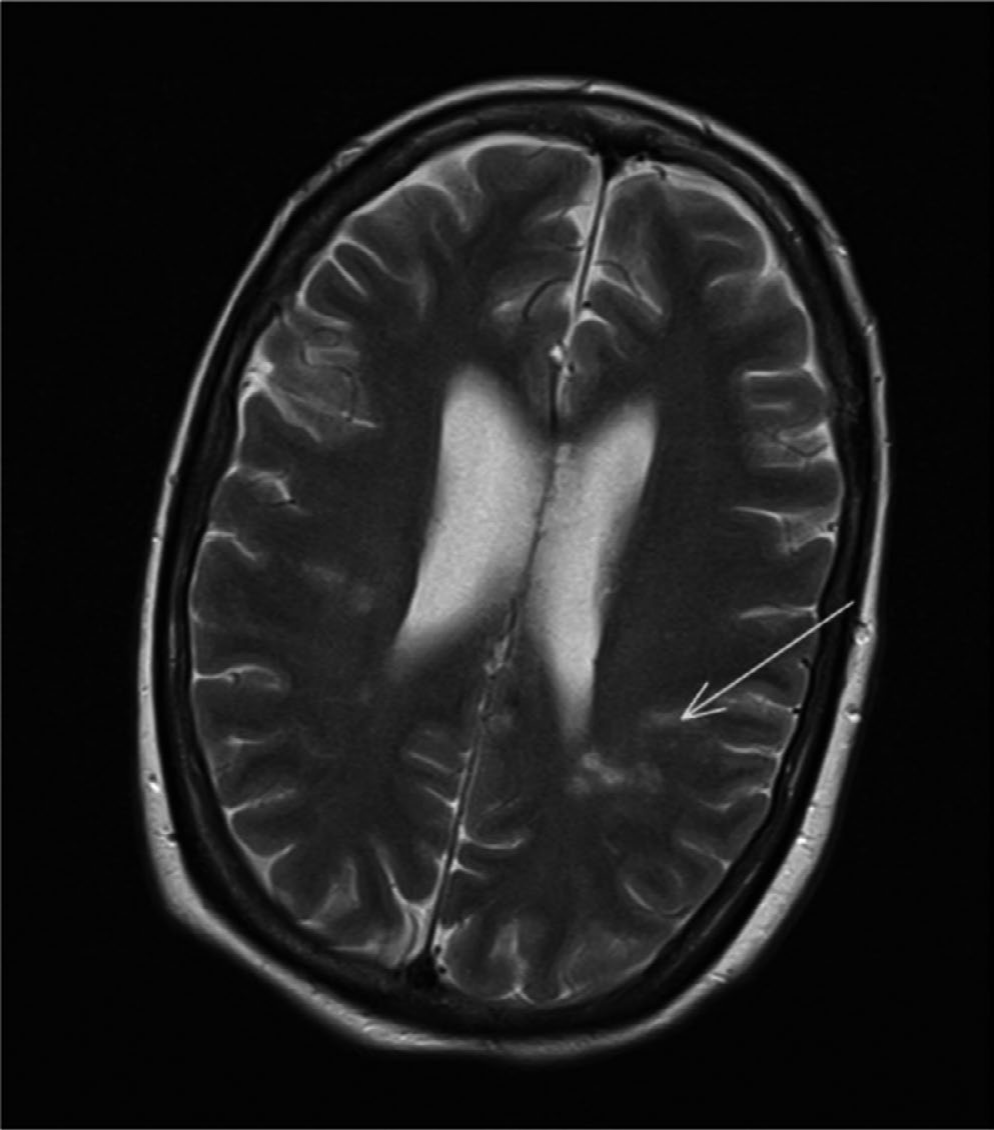
MRI head with contrast: axial T2 image. Arrow shows area of increased signal in middle cerebral artery territory indicating acute infarction

The parietal lobe stroke was presumed to be an embolic event secondary to the left atrial tumor. Debris from the left pulmonary vein removed during surgery had the appearance of reactive blood clot. Magnetic resonance imaging (MRI) of the brain following surgery showed areas of diffusion restriction in the left middle cerebral artery (Figure 2) and posterior cerebral artery territories suggestive of embolic phenomena not visualized on CT scanning.

## 3 DISCUSSION

The advantages of using FICE routinely in the intensive care unit are well recognized and are highlighted by this case. In this instance, information gleaned directly from the FICE scan led to diagnosis and definitive treatment, potentially avoiding a delay in diagnosis and further life‐threatening embolic events. Left atrial tumors have high embolic potential,^5^ and embolization to the cerebral arteries is common. Emboli are also often found in the pulmonary artery and peripheral vasculature.^6^

Although the remit of FICE is to make a basic assessment of cardiac function, significant cardiac pathology can be identified by the competent practitioner. Echocardiography is playing an increasing role in the management of critical care patients, and given the finite availability of BSE accredited practitioners, FICE plays an important role in bridging this clinical service gap.

In a single‐center study of the use of FICE in critical care, 68% of FICE scans showed previously unknown findings,^7^ illustrating its value in clinical decision making. In the majority of cases, this was left ventricular dysfunction or hypovolemia. Identifying these common critical care issues is what the FICE protocol was designed for, yet in the process of training to identify these common issues, practitioners can also develop their understanding of cardiac anatomy to a point that recognition of more complex pathology can be achieved accurately enough to trigger additional investigation. This is an area of FICE accreditation which has not been fully explored—likely because of concerns about encroaching on the remit of those with advanced skills in TTE and more importantly concerns that FICE images will be used to incorrectly diagnose conditions beyond the scope of the protocol. Of course, those carrying out FICE scans need to understand its limitations and obtain more specialist investigations if concerned about scan findings in a patient. Importantly, the use of FICE is not in replacing formal echocardiography but to ascertain some basic information or as a bridge to formal echocardiography.

The case we have reported here also highlights the argument for carrying out a FICE scan on every critical care patient as part of their admission investigations. Had this lady had a scan on admission, she potentially would have had her operation 24 hours earlier. Thus, routine FICE scans on admission could have a direct and positive impact upon time to definitive management for complex patients. Indirectly, this can reduce time to extubation and discharge from intensive care, which as we know affects mortality and morbidity in critical care patients. This is in addition to reducing pressure on other limited clinical resources. The flipside to routine FICE scanning is increased incidental findings potentially leading to over‐investigation.

Internationally, it is accepted that “basic” level echocardiography is a skill which all critical care practitioners should possess.^8^ FICE accreditation is an accessible means of achieving this aim. FICE already appeals to critical care trainees as they can achieve the accreditation in a relatively short timeframe. FICE scans offer wider benefits for patient care and management of clinical resources. Routine FICE scans for all critical care admissions would also offer a greater number of training opportunities in echocardiography for critical care practitioners. More crucially, routine FICE scans on admission would increase diagnostic yield particularly in more complex patients as this case demonstrates.

## CONFLICT OF INTEREST

The authors declare that they have no competing interests.

## AUTHOR'S CONTRIBUTIONS

JM and LA: designed the report and completed the manuscript. JM, GW, and AR: performed imaging. GW, AR, and LA: revised the manuscript. All authors read and approved the final manuscript.
